# Non-invasive Estimation of the Intracranial Pressure Waveform from the Central Arterial Blood Pressure Waveform in Idiopathic Normal Pressure Hydrocephalus Patients

**DOI:** 10.1038/s41598-018-23142-7

**Published:** 2018-03-16

**Authors:** Karen Brastad Evensen, Michael O’Rourke, Fabrice Prieur, Sverre Holm, Per Kristian Eide

**Affiliations:** 1 Department of Informatics, University of Oslo, Oslo, Norway; 2 Department of Neurosurgery, Oslo University Hospital - Rikshospitalet, Oslo, Norway; 3 Department of Cardiology, St Vincent’s Clinic, University of New South Wales/VCCRI, Sydney, Australia; 4 Institute of Clinical Medicine, Faculty of Medicine, University of Oslo, Oslo, Norway

## Abstract

This study explored the hypothesis that the central aortic blood pressure (BP) waveform may be used for non-invasive estimation of the intracranial pressure (ICP) waveform. Simultaneous invasive ICP and radial artery BP waveforms were measured in 29 individuals with idiopathic normal pressure hydrocephalus (iNPH). The central aortic BP waveforms were estimated from the radial artery BP waveforms using the SphygmoCor system. For each individual, a transfer function estimate between the central aortic BP and the invasive ICP waveforms was found (Intra-patient approach). Thereafter, the transfer function estimate that gave the best fit was chosen and applied to the other individuals (Inter-patient approach). To validate the results, ICP waveform parameters were calculated for the estimates and the measured golden standard. For the Intra-patient approach, the mean absolute difference in invasive versus non-invasive mean ICP wave amplitude was 1.9 ± 1.0 mmHg among the 29 individuals. Correspondingly, the Inter-patient approach resulted in a mean absolute difference of 1.6 ± 1.0 mmHg for the 29 individuals. This method gave a fairly good estimate of the wave for about a third of the individuals, but the variability is quite large. This approach is therefore not a reliable method for use in clinical patient management.

## Introduction

Monitoring of intracranial pressure (ICP) has an important role in surveillance and diagnostics of patients with brain injury of various causes^1^. Current clinical methods for monitoring ICP are invasive, and thus require a hole to be drilled in the skull in order to place a device within the brain parenchyma. This procedure imposes risks of severe complications such as intracranial bleeds in 1–2% of patients^2^, which combined with the complexity and invasiveness of the procedure limits its clinical applicability. As a result, ICP monitoring is only performed on a limited patient selection, although being advantageous for a much larger group.

Despite the apparent benefits of non-invasive ICP monitoring, none of the previously reported methods are sufficiently accurate for routine clinical use^3–6^, although the use of transcranial acoustic signals has shown some promise^7^. Currently monitoring of mean ICP levels is the established standard approach, but ICP waveform analysis may provide additional information about intracranial compensatory reserve capacity (i.e. intracranial compliance)^8–10^. Non-invasive prediction of the ICP waveform may therefore have significant clinical value.

One approach for non-invasive ICP monitoring has been to estimate ICP from radial artery blood pressure (BP) measurements, either solely based on radial artery BP, or in conjunction with blood velocity measurements^11–13^. This is an appealing approach as radial artery BP is routinely measured in clinical setting. Both the radial artery BP waves and the ICP waves are created from BP waves induced by the cardiac beat contractions. Each cardiac beat contraction produces an ICP wave that passes through the intracranial compartment. It has therefore been proposed that the central aortic BP waveform is a better source for nICP estimation than the radial artery BP waveform. This was supported by a previously conducted preliminary study that reported that the central aortic BP waveform compared better with the ICP waveform than the radial artery BP retrieved from radial artery measurements^14^. The authors reported that the central aortic BP waveform was almost identical to the ICP waveform during the period of systole, and that the augmentation index was similar to that of ICP. The radial artery BP and ICP waveforms were notably more different.

The present study was undertaken to examine this hypothesis further and to study how the ICP waveform associates with the central aortic BP waveform. The central aortic BP waveforms were estimated from the radial artery BP waveform using the SphygmoCor system^15^. Two approaches were used, one Intra-patient approach and one Inter-patient approach. The first approach was to generate a transfer function estimate from the central aortic BP waveform to the invasive ICP waveform for each patient. This was done to investigate the potential of the method. The second approach was to utilize the transfer function estimate from the Intra-patient approach that gave the highest cross correlation between the non-invasive ICP estimate and the invasively measured ICP waveforms on the total cohort of individuals. The first approach provides important information about the possibility of using the central aortic BP waveforms as a source for non-invasive ICP estimation, while the second approach has potential clinical value.

## Materials and Methods

### Patient material and ethical approval

To validate whether the central aortic BP waveforms can be used to estimate the ICP waveform non-invasively, a set of simultaneous ICP and radial artery BP waveforms from 29 patients with idiopathic normal pressure hydrocephalus (iNPH) was retrieved.

The ICP and radial artery BP waveforms had been obtained as part of a study approved by the Regional Ethics Committee, REK South-East (approval no. 07362) and by the hospital authority (approval no. 07/5870). The study protocol was in accordance with relevant guidelines and regulations and inclusion was by written and oral informed consent. The enrolled subjects were patients admitted to the Department of Neurosurgery, Oslo University Hospital - Rikshospitalet, from October 2008 to January 2009, for whom continuous invasive ICP monitoring was a part of their clinical work-up. The ICP levels measured were not used as an enrolment or exclusion criterion, and inclusion in the study did not influence the patient management. The continuous ICP and artery BP recordings were stored as anonymous raw data files to be analysed at a later stage.

### Measurements of radial artery BP and ICP waveforms

Overnight invasive monitoring of radial artery BP and ICP was performed as described in^16^. The radial artery BP was continuously measured in the right radial artery using a Truwave PX-600F Pressure Monitoring Set (Edwards Life sciences LLC, Irvine, CA). The radial artery BP sensor was placed at the level of the heart.

Simultaneously with the BP measurements, ICP was continuously monitored using a solid ICP sensor (Codman MicroSensor^TM^, Johnson & Johnson, Raynham, MA, USA), which was introduced 1–2 cm into the frontal brain parenchyma through a small burr hole and a minimal opening in the dura, as described in^17^.

Both the radial artery BP and ICP waveforms were sampled at 200 Hz, ensuring a sufficient sampling rate^18^. The data was digitized using the Sensometrics^®^ Pressure Logger (dPCom AS, Oslo, Norway) and analysed using Sensometrics^®^ software (dPCom AS, Oslo, Norway).

Implantation of an ICP sensor may give some cerebrospinal fluid (CSF) leakage. The first two hours of monitoring were therefore omitted for all individuals.

### Estimation of the central aortic BP waveforms from the radial artery BP waveforms

The central aortic BP waveforms were estimated from the radial artery BP waveforms using the SphygmoCor system (SphygmoCor®; AtCor Medical, West Ryde, NSW, Australia). For each patient, a raw data file with the time series of the central aortic BP and ICP waveforms were obtained, both having identical time reference.

### Transfer function estimation

In^19^ Gao *et al*. presents a patient specific transfer function based on a model consisting of tubes, travel time and reflection coefficients. In this paper the complex piping system from the central aorta to the brain is modelled as a black box using signal processing and spectral analysis.

The initial assumption is a linear, time-invariant system, where the output *y* is connected to the input *x* as $y=h\ast x$, where * denotes a linear convolution and *h* the system’s impulse response. The Fourier Transform

1 $$H(f)=\frac{Y(f)}{X(f)},$$

produces a direct frequency domain description of the system properties through the transfer function *H*(*f*). This allows for simpler signal processing than their corresponding signals in the time domain. When the transfer function is unknown, a transfer function estimate $\hat{H}(f)$ can be found from

2 $$\hat{H}(f)=\frac{{P}_{xy}(f)}{{P}_{xx}(f)},$$

for the case of a single-input/single-output system^20^. In this equation *P*_*xx*_(*f*) is the power spectral density of the input signal and *P*_*xy*_(*f*) the cross power spectral density function of *x* and *y*. The latter is given as

3 $${P}_{xy}(f)=\sum _{m=-\infty }^{\infty }{R}_{xy}(m){e}^{-j2\pi fm},$$

where *j* denotes the imaginary number,$E\{\cdot \}$ the expected value operator and *R*_*xy*_(*m*) the cross-correlation sequence

4 $${R}_{xy}(m)=E\{{x}_{n+m}{{y}_{n}}^{\ast }\}.$$

The power spectral density *P*_*xx*_(*f*) is found by using the auto-correlation variant of equation (3), where *R*_*xy*_(*m*) is replaced by *R*_*xx*_(*m*).

**Figure 1:**
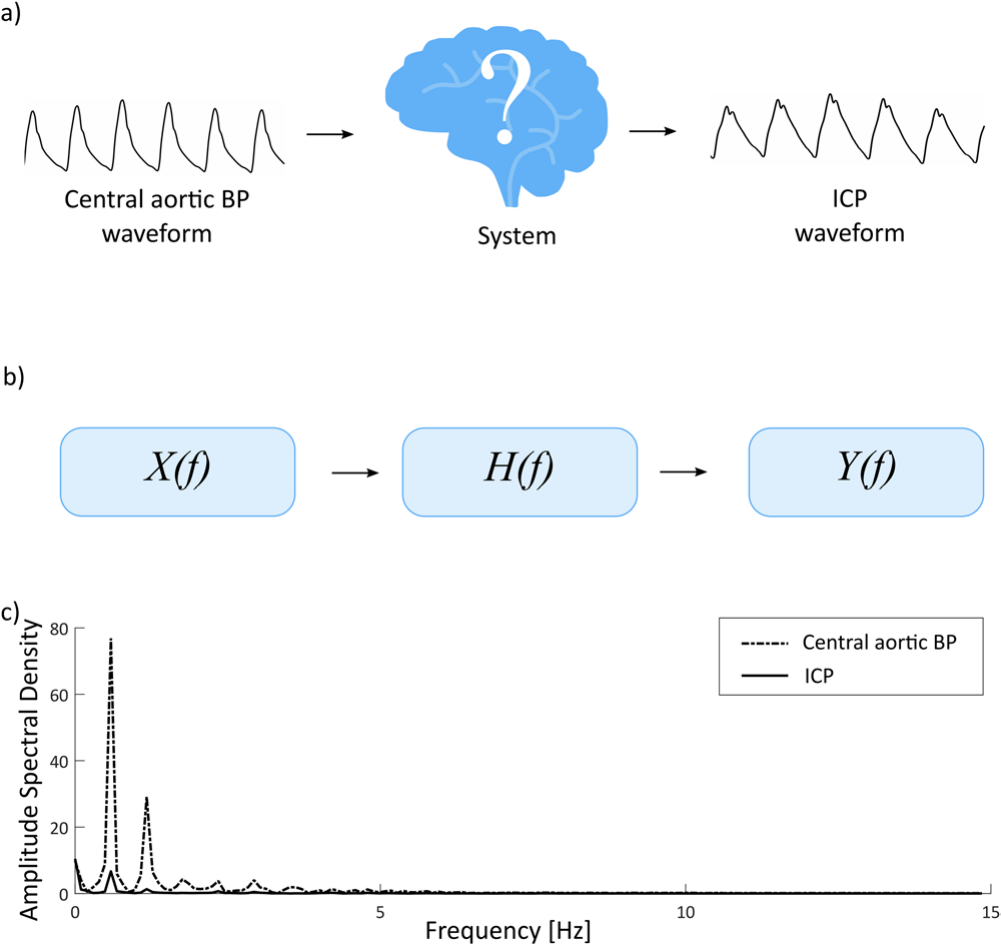
Non-invasive estimation of ICP waveforms from central aortic BP waveforms. (**a**) In this study, central aortic BP waveforms were used as input for estimation of non-invasive ICP signals, when the system from the heart to the cranium was said to be unknown. (**b**) A system description in the frequency domain is found from the transfer function $H(f)$. (**c**) An estimate for the system is found based on the power spectral density of the central aortic BP waveforms (dotted line) and ICP waveforms (continuous line).

The most direct application of single-input/single-output relationships like this is to estimate the system frequency response function, or transfer function, based on measured input/output data. In this study, the preliminary objective was to determine the transfer function from the central aortic BP signals to the ICP signals, and then use this for future non-invasive ICP estimation. The discrete time series of the central aortic BP signal *x*[*n*], was used as input with *n*=0, …, *N*–1, and $N$ denoting the total number of time samples. The equivalently sampled invasive ICP measurements, here denoted *y*[*n*], were used as output. The basic concept is illustrated in Fig. 1. As the transfer functions established in our study are estimated from the estimates of central aortic BP and measured ICP, height and weight are implicitly compensated for. With regard to the present data, linearity was assumed since ICP is in low range (mean ICP 1.8 ± 3.6 mmHg). In another situation with very high ICP, non-linear associations might be expected.

### Transfer function estimation: Intra-patient approach

In order to establish patient specific transfer function estimates, an individual $\hat{H}(f)$ was found for each patient using MATLAB’s predefined *tfestimate* function. This function uses Welch’s averaged periodogram method to minimize variance (MATLAB and Statistics Toolbox Release 2016a, The MathWorks Inc., Natick, Massachusetts, USA). A total of one hour of data was used for the transfer function estimation. The one hour was divided into six second windows with 50% overlap in order to find a representative average.

The first hour of data after midnight was used in all cases, except for patient ID 7, where measurements had not been performed around midnight. In this case the data between 02.00 and 03.00 a.m. was used. The reason for selecting this point in time was that all patients were in bed, and therefore the pressure measurements were most standardized. The mean was removed from the input and output series in order to ensure that the transfer function analysis would not be influenced by fluctuations in mean ICP level and to avoid side lobe leakage^21^. After establishing the patient specific transfer function estimates, all data above 15 Hz were zeroed, as prior studies have shown that the significant harmonic content of the central aortic BP waveforms is contained within 0 and 15 Hz^15^.

**Figure 2:**
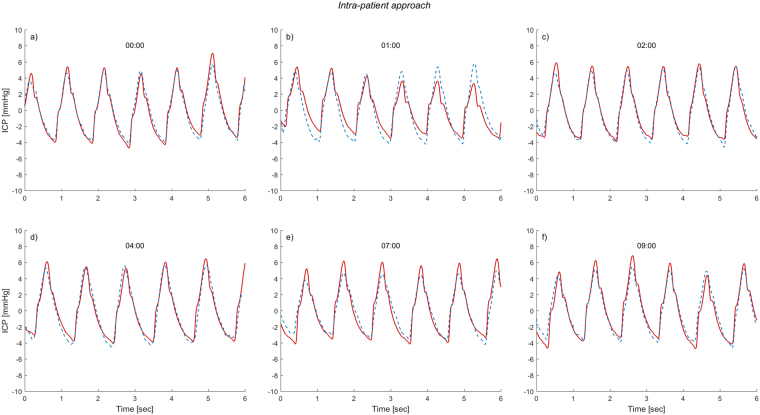
The non-invasive ICP waveform estimated from the Intra-patient approach superimposed on the invasive ICP waveform. For six different time windows of six second duration, the invasive ICP raw signal (continuous red line) is shown superimposed on the estimated non-invasive ICP signal (interrupted blue line) for patient 20. The estimate is established with the Intra-patient approach and shown for the time points (**a**) 00:00, (**b**) 1 hour after 00:00, (**c**) 2 hours after 00:00, (**d**) 4 hours after 00:00, (**e**) 7 hours after 00:00, and (**f**) 9 hours after 00:00. For visual comparison, the estimated ICP signal is time-shifted to match the invasive ICP signal for each time window.

The transfer function estimates established for each individual was then applied to the remaining part of the recording using equation (1), and thus giving an estimated non-invasive ICP signal for the specific patient. Figure 2. shows an example of the results where the non-invasively estimated ICP waveform is plotted together with the invasively measured ICP waveform. The figure shows six different time windows for one patient.

### Transfer function estimation: Inter-patient approach

**Figure 3:**
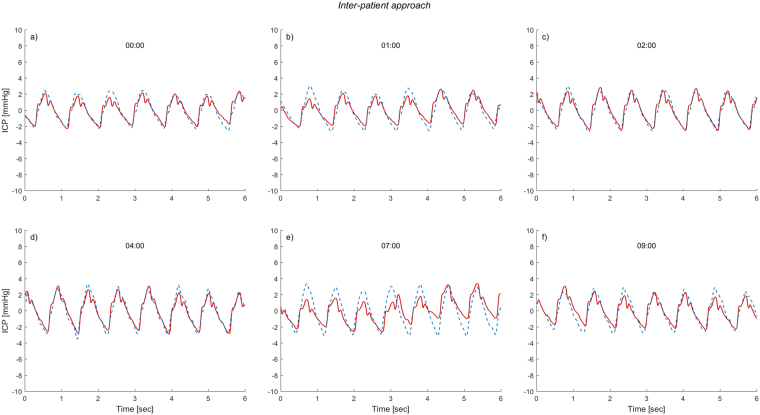
The non-invasive ICP waveform estimated from the Inter-patient approach superimposed on the invasive ICP waveform. For six different time windows of six second duration, the invasive ICP raw signal (continuous red line) superimposed on the estimated non-invasive ICP signal (interrupted blue line) are shown for one patient, established with the Inter-patient approach. The results are shown for the time points (**a**) 00:00, (**b**) 1 hour after 00:00, (**c**) 2 hours after 00:00, (**d**) 4 hours after 00:00, (**e**) 7 hours after 00:00, and (**f**) 9 hours after 00:00. For visual comparison the estimated non-invasive ICP signal is time-shifted to match the invasive ICP signal for each time window.

The transfer function estimate, which gave the best cross-correlation between the non-invasive and invasive ICP signal ($\hat{H}(f)$ for patient ID 20), was then applied to the central aortic BP waveforms for all other patients using equation (1). This resulted in non-invasive ICP time domain estimates for the total cohort of patients. All available data from midnight until the end of the measurements were utilised. An example showing the resulting estimated non-invasive ICP waveform together with the invasively measured ICP waveforms is shown in Fig. 3. The figure shows six different time windows where the transfer function estimate found for patient ID 20 is applied to patient ID 8.

### Comparison of measured and estimated ICP waveforms

In order to validate the output of the Intra-patient and the Inter-patient approaches, the non-invasive ICP estimates were compared to the invasive ICP measurements using a time-domain method previously described by Eide in^17^. According to this method, the cardiac-induced waves were identified by their beginning and ending diastolic minimum pressures and systolic maximum pressures. For each cardiac-beat–induced ICP wave, the pulse amplitude (dP; pressure difference from diastolic minimum pressure to systolic maximum pressure), rise time (dT; time difference from diastolic minimum pressure to systolic maximum pressure) and rise time coefficient (RTC, dP/dT) were determined. Further, the ICP waveform indices such as the mean wave amplitude (MWA), the mean wave rise time (MWRT) and the mean wave rise time coefficient (MWRTC) were computed for subsequent six-second (6-sec) time windows. The mean wave amplitude (MWA) is a type of time averaging of the pulse amplitude (dP). For detailed description of the MWA parameter, see^17^.

Only 6-sec time windows containing minimum four cardiac beat induced waves were considered to be of good quality and were used for the present analysis. The software also identified artefact waves due to noise in the pressure signal caused by patient movement, sensor movement or dysfunction. Such artefact waves were consequently omitted from the analysis. For each 6-sec time window of non-invasive and invasive ICP recordings, the differences between estimated and measured MWA, RT and RTC were determined.

In the diagnostic assessment of patients with idiopathic normal pressure hydrocephalus (iNPH), the invasively measured MWA (iMWA) is used to select patients for surgery. Based on previous studies, an upper normal threshold of iMWA has been determined^22^. To test the clinical utility of central aortic BP-derived non-invasive MWA (nMWA), the predictive values of non-invasive MWA versus invasive MWA were tested.

### Statistics

The statistical analyses were performed using the SPSS software version 22 (IBM Corporation, Armonk, NY). Statistical significance was accepted at the 0.05 level.

## Results

### Patient material

A total of 29 patients were included in the study. Supplementary Table 1 presents demographic data of the patients, as well as basic physiological data about mean BP from radial artery, mean ICP and mean cerebral perfusion pressure (CPP).

### Validation of the Intra-patient and Inter-patient approaches

**Figure 4:**
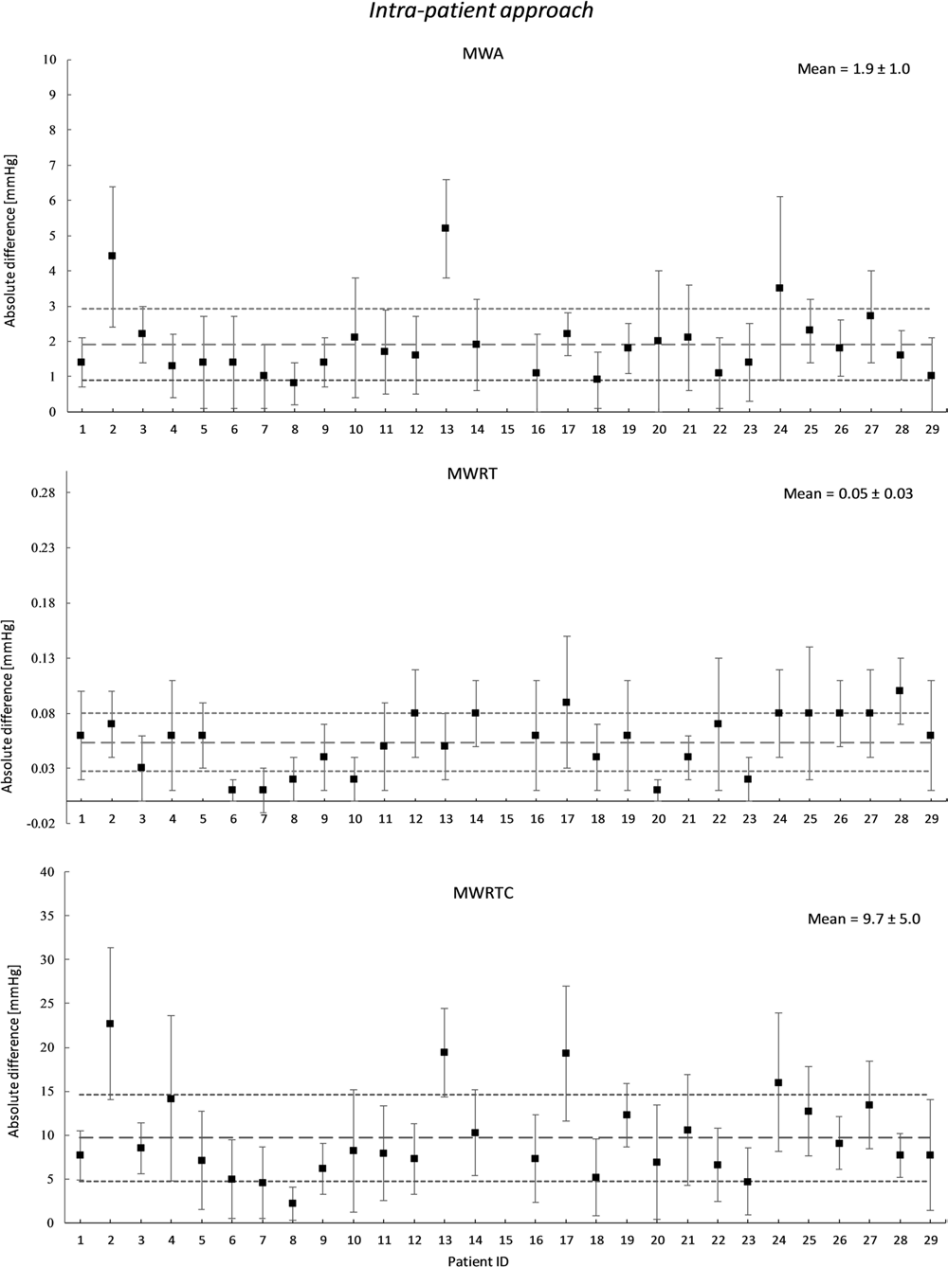
Differences in absolute pulsatile pressure parameters between measured ICP and estimated pulsatile ICP according to Intra-patient approach. The averaged absolute difference between the measured ICP and the estimated ICP is shown for the Intra-patient approach. This is shown for each time domain waveform parameter for each patient ID, with the patient specific standard deviation illustrated as error bars. The total mean for the patient cohort together with its standard deviation is illustrated with dotted lines.

The differences in absolute pulsatile pressure parameters between measured ICP and estimated pulsatile ICP according to the Intra-patient approach are presented in Fig. 4. In addition, Supplementary Table 2 presents the ICP waveform parameters retrieved from the measured invasive ICP signal to the left, and the absolute differences in ICP waveform parameters estimated according to the Intra-patient approach to the right. For the total cohort of 29 individuals, the mean absolute difference in MWA was 1.9 ± 1.0 mmHg. The difference in MWA was <1.0 mmHg for 2 of 29 patients, and the absolute differences in MWRT and MWRTC were 0.05 ± 0.03 sec and 9.7 ± 5.0 mmHg/sec, respectively (Fig. 4; Suppl. Table 2). The measured and estimated ICP waveform parameters were compared in 137,512 6-sec time windows for the 29 individuals (Suppl. Table 2).

**Figure 5:**
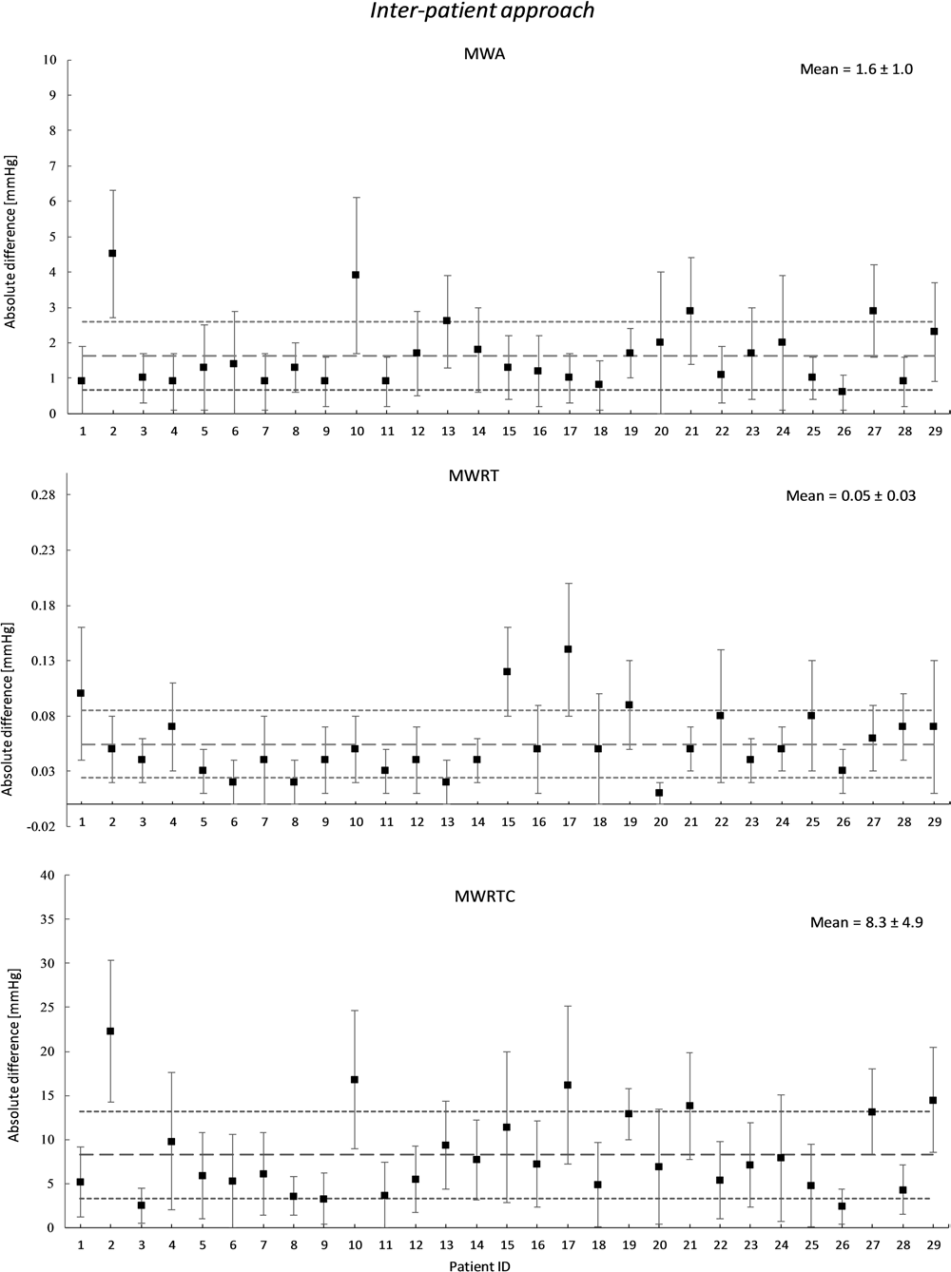
Differences in absolute pulsatile pressure parameters between measured ICP and estimated pulsatile ICP according to Inter-patient approach. The averaged absolute difference between the measured ICP and the estimated ICP is shown for the Inter-patient approach. This is shown for each time domain waveform parameter for each patient ID, with the patient specific standard deviation illustrated as error bars. The total mean for the patient cohort together with its standard deviation is illustrated with the dotted lines.

Figure 5 presents the differences in absolute pulsatile pressure parameters between measured ICP and estimated pulsatile ICP according to Inter-patient approach. Supplementary Table 3 provides the absolute differences in pulsatile ICP parameters estimated non-invasively using the Inter-patient (shown to the right). The averaged absolute difference in MWA was found to be 1.6 ± 1.0 mmHg for the total cohort of 29 individuals. Furthermore, the absolute difference in MWA was <1 mmHg in 8 out of 29 patient recordings. The averaged absolute differences in MWRT and MWRTC were 0.05 ± 0.03 sec and 7.0 ± 6.1 mmHg/sec respectively (Fig. 5; Suppl. Table 3). These results indicate that the Inter-patient approach produces a better result than the Intra-patient approach.

For both the Intra- and Inter-patient approaches, Table 1 presents the percentage of 6-sec time windows wherein absolute differences in MWA were <0.5 mmHg or <1.0 mmHg. The Inter-patient approach seemed to perform best; on average an absolute difference in MWA <0.5 mmHg was observed in 22.2% of 6-sec time windows, while the average absolute difference in MWA <1.0 mmHg was seen in 42.1% of 6-sec time windows. For example, a difference in absolute MWA <0.5 mmHg in >30% of 6-sec time windows was observed in 9 of 29 individuals (PatIDs 1, 4, 5, 7, 9, 11, 18, 26 and 28).

**Table 1:** Percentage of individual 6-s observations with difference in MWA either <0.5 mmHg or <1.0 mmHg for the two approaches.

PatID	Intra-patient approach		Inter-patient approach	
	Difference in MWA <0.5 mmHg: Percentage of 6-s observations	Difference in MWA <1.0 mmHg: Percentage of 6-s observations	Difference in MWA <0.5 mmHg: Percentage of 6-s observations	Difference in MWA <1.0 mmHg: Percentage of 6-s observations
1	11	30	47	76
2	1	2	0	1
3	0	4	24	52
4	19	44	32	61
5	30	53	31	56
6	23	47	26	48
7	38	61	38	65
8	35	66	11	32
9	4	34	42	62
10	15	30	3	8
11	16	33	32	62
12	12	31	23	36
13	0	0	5	12
14	12	26	13	28
15			24	46
16	32	57	21	51
17	0	0	28	58
18	34	67	43	67
19	2	14	0	13
20	19	38	19	38
21	12	25	1	5
22	32	60	27	49
23	22	42	19	36
24	6	14	22	39
25	0	3	22	49
26	4	14	51	83
27	4	9	3	7
28	5	22	34	68
29	36	64	4	12
**AVG ± STD**	**15.1 ± 13.0**	**31.8 ± 21.8**	**22.2 ± 14.7**	**42.1 ± 23.2**

### Predictive ability of estimated pulsatile non-invasive ICP

The ability of the non-invasive ICP estimates to predict MWA, and thereby the clinically important thresholds of MWA (</≥ 4 mmHg), are presented in Table 2. For both the Inter-patient and the Intra-patient approach, the Negative Predictive Value (NPV) of the non-invasive MWA was 42%.

**Table 2:** Predictive ability of Intra-patient and Inter-patient approaches for MWA threshold of 4 mmHg.

Transfer function (Intra-patient approach)	Number	Test results
Measured/Estimate ≥4 mmHg	9	Sensitivity 45%
Measured /Estimate <4 mmHg	8	Specificity 100%
Measured ≥4/Estimate<4	11	PPV 100%
Measured <4/Estimate≥4	0	NPV 42%
Measured /Estimate ≥4 mmHg	14	Sensitivity 67%
Measured /Estimate<4 mmHg	5	Specificity 63%
Measured ≥4/Estimate<4	7	PPV 82%
Measured <4/Estimate ≥4	3	NPV 42%

PPV: Positive predictive value. NPV: Negative predictive value.

### Impact of other factors on the results

**Figure 6:**
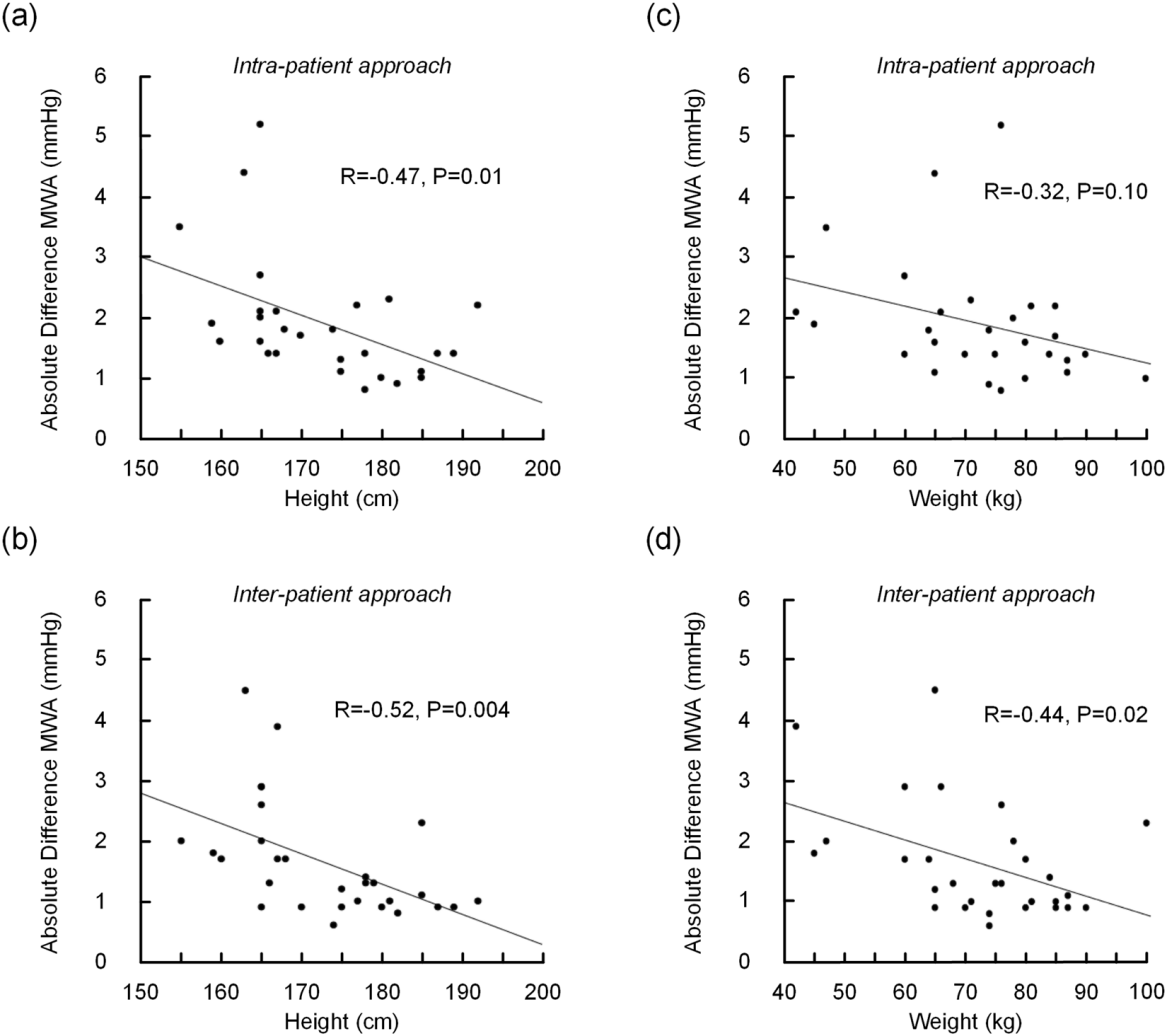
Association between height and weight with absolute differences in MWA, as estimated from the Intra- and Inter-patient approaches. The associations between the height of the patients and the absolute difference in MWA between measured and estimated ICP waveforms were determined according to (**a**) the Intra-patient approach and (**b**) the Inter-patient approach. Further, the association between the weight of the patients and the absolute difference in MWA between measured and estimated ICP waveforms were determined according to (**c**) the Intra-patient approach and (**d**) the Inter-patient approach. For each plot the fit line and the Pearson correlation coefficient (R) with significance level is presented.

We also examined whether other factors, such as physical parameters, affected the quality of the non-invasive ICP estimates. Both the height and weight of the patient affected the differences in MWA determined according to the Intra-patient approach. Patient height also impacted the non-invasive ICP results for the Inter-patient approach (Fig. 6). However, there were no evident similarities between the height and weight for patient ID 20 and the height and weight for the patient IDs with the best non-invasive ICP estimates. Other factors such as age and levels of mean BP or mean ICP did not affect the results. In particular, we found no correlation between the estimated MWA scores and the static pressure scores (i.e. mean BP, mean ICP, mean CPP).

## Discussion

The presented data show some promise regarding the ability of central aortic BP waveforms to non-invasively estimate the ICP waveform. The central aortic BP can under certain conditions serve as a source for non-invasive ICP monitoring, but the results are not consistent.

To estimate non-invasive pressure waveforms from invasive measurements using transfer functions is not new in literature. One example is the SphygmoCor system (SphygmoCor®; AtCor Medical, West Ryde, NSW, Australia) currently used in clinic, where the central aortic BP waveforms are estimated from the radial artery BP waveforms by a generalized, and population averaged, transfer function. This generalized transfer function was developed by Karamanoglu and O’Rourke *et al*.^23^ and has been shown to give central aortic BP estimates that are in good agreement with invasive central aortic BP measurements in cardiac catheterization patients^24,25^. Gao *et al*.^19^ took the concept further, and presented a simple model for an adaptive transfer function which produces comparable results to the SphygmoCor system. This transfer function also relies on radial artery BP waveform measurements, but in addition some patient specific physiological parameters were included. The latter represents the adaptiveness of the method and decides the travel time and reflection coefficient in the model.

In this study, the hypothesis was that the ICP waveform can be decided non-invasively from the central aortic BP waveform using a transfer function. The system, and hence the transfer function estimate for each patient, is expected to be constant. Non-invasive ICP estimation should thereby be possible on patients where invasive ICP measurements already has been done. This was denoted the Intra-patient approach in this paper, which alone will have very limited clinical potential, as it relies on patient specific training data requiring invasive ICP measurements.

When remembering Gao *et al*.’s^19^ approach to transfer function estimation, one would expect different patients to have similar transfer functions if similar biological parameters such as age, height, weight and mean ICP coincide. The first three are sizes available for the physician and could thereby be used as input when selecting a pre-determined transfer function from an already established database. This would give an almost completely non-invasive patient specific ICP estimate, where the invasiveness is limited to an intra-arterial line for radial artery BP measurements. If successful, this approach (the Inter-patient approach) would have significant clinical value.

The comparison of the non-invasive ICP estimates and the invasive ICP signals for the Intra-patient approach is shown in Fig. 4, which illustrates variability of the quality of the estimated ICP signals. Whereas the approach works for some patients, it gives a very non-informative result for others. When the differences in the most important clinical parameter MWA was explored, it was found to be <1 mmHg for 2 of 29 patients (Suppl. Table 2).

One of the assumptions made in the initial determination of the transfer function estimates was that the system had to be linear and constant for the estimates to be valid. It was expected that this would be a relatively good approximation for the case of the Intra-patient approach, but as evident in Table 1 and Supplementary Table 2, this does not match the presented results. The varying quality of the results indicates that the initial assumptions are not applicable for the entire cohort of 29 patients, but rather a subgroup. This is a clear limitation and further studies are needed to explore possible reasons. One confounding factor that might affect the results is variability in pressure cerebrovascular auto-regulation. Variability in cardiovascular co-morbidity might be another factor. It should also be mentioned, that although the SphygmoCor system has been shown to give good central aortic BP estimates, they are estimates, and thus an additional source of uncertainty.

The present patient cohort only included individuals with the condition iNPH, which is a neurodegenerative hydrocephalic disease in adults. One advantage with this cohort is that ICP monitoring was not combined with drainage of CSF. The ICP waveforms of this particular patient cohort do not present with particular characteristics as compared to other patient groups, e.g. children^26^ or individuals managed for stroke within the intensive care unit^27^. Accordingly, there are no particular features with these ICP recordings making them more or less useful for validation of a non-invasive ICP estimation approach. This is, however, the only condition this method has been applied to, and the results presented in this study are not necessarily generalizable to other patient populations or normal human subjects.

Validation of the non-invasive ICP estimates found from the Inter-patient approach showed a mean absolute difference in MWA of 1.6 mmHg and a mean absolute difference in MWRTC of 8.3 mmHg/sec. These results are better than for the Intra-patient approach. The differences between the two approaches at the individual level are presented in Supplementary Tables 2 and 3. For some individuals, the invasive and non-invasive ICP waveform parameters were rather comparable. In the clinic, however, the invasive MWA is used as a significant parameter to tailor management of neurosurgical patients. Depending on the reason for measuring invasive MWA, the upper normal threshold is about 4 mmHg^22,26,27^. The only effective treatment of iNPH is shunt surgery. For these patients, clinical benefit of surgery was found in 9/10 individuals with MWA above threshold, while only in 1/10 with MWA below threshold. The threshold was defined as an average MWA of 4 mmHg with more than 5 mmHg in at least 10% of the recording time^22,28^. As detailed in Tables 1 and 2, the estimated non-invasive ICP signals did not reliably reproduce the invasive MWA thresholds. The negative predictive values were 42% for both the Intra- and Inter-patient approaches, while the positive predictive values were 100% and 82%, respectively (Table 2). These results are not good enough for central aortic BP-derived non-invasive ICP estimates to be used in clinical setting at present.

In the past, several attempts have been made to estimate non-invasive ICP based on invasive central aortic BP and Doppler-based cerebral blood flow measurements^11–13,29^. The approaches presented in this paper differ by relying on radial artery BP and the estimated central aortic BP waveforms alone. Only having to collect radial artery BP and basic physiological parameters such as height/weight would considerably ease the situation for the physician, and would therefore be of significant value. In a preliminary study it is suggested that the central aortic BP waveform may be used to estimate the ICP signals non-invasively^14^. The current study extends on this by incorporating a rather large patient cohort. Unlike previous studies, the present study also focuses on pulsatile ICP instead of mean ICP.

When investigating the non-invasive ICP estimates, it was found that the results were impacted by height and weight, while other factors such as age and level of mean radial artery BP and mean ICP had no significant impact. The fact that height and weight influenced the results complies with what was expected for the Inter-patient approach. We would especially expect better results for the patients with similar properties as patient ID 20. We would then have a similar system, and expect to have similar transfer functions, hence producing better results. This was however not the case, indicating that a simple tube length/reflection coefficient description will not be sufficient when estimating ICP non-invasively from radial artery BP. The variability of the Intra-patient approach points towards a much more complex mechanism than a simple linear system linking central aortic BP to ICP. The relevance of height and weight for the Intra-patient approach was surprising, and clearly indicates that the patient’s height and weight in general affected the successfulness of the approach.

The diagnosis of the patient, and co-morbidity such as cardiovascular disease, may influence the patient’s system, and thereby its transfer function estimate and resulting non-invasive ICP signal. When further investigations are made this should be taken into consideration.

## Conclusions

In the present study, it has been investigated whether central aortic BP signals estimated from radial artery BP signals can be used to non-invasively predict the pulsatile ICP waveform. Patient specific transfer function estimations from the central aortic BP signals to invasively measured ICP signals has been found for a total cohort of 29 patients. The patient specific transfer functions were further utilized to find individual ICP estimates for each patient. A time domain analysis of the estimated ICP compared to the invasive ICP signals found that the estimates correctly predicted the most important clinical parameter MWA in about 2 of 29 cases. This indicates that the method has some potential, but that there are large uncertainties.

For the method to have significant clinical value it should be possible to estimate ICP signals without first measuring invasive ICP signals. This was achieved by using the transfer function estimate that gave the best cross-correlation between the estimated ICP and measured ICP on the total cohort of 29 individuals. The resulting ICP estimates correctly predicted the MWA parameter within the necessary range in 8 out of 29 cases. However, they did not reproduce the invasive MWA threshold. As the quality of the results are too varying, these results are inadequate for central aortic BP-derived non-invasive ICP estimates to be used in the clinical setting. However, the method shows some promise regarding utility of the central aortic BP waveform to predict the ICP waveform. The assumption of a linear system linking central aortic BP to ICP seems to be too simplistic and the model should be expanded to incorporate more of the complexity of the system. Further studies should therefore be performed to determine the future clinical possibilities of this approach.

## Electronic supplementary material

Supplementary Information

## References

[1] Czosnyka M (2004). Monitoring and interpretation of intracranial pressure. J. Neurol. Neurosurg. Psychiatry.

[2] Binz DD, Toussaint LG, Friedman JA (2009). Hemorrhagic complications of ventriculostomy placement: a meta-analysis. Neurocritical care.

[3] Popovic D, Lee KM, Noninvasive S (2009). monitoring of intracranial pressure. Recent Patents Biomed Engineer.

[4] Zhang, X. *et al*. Invasive and noninvasive means of measuring intracranial pressure: a review. *Physiol. Meas*. (2017). 10.1088/1361-6579/aa7256 28489610

[5] Robba C (2016). Non-invasive assessment of intracranial pressure. Acta Neurol. Scand..

[6] Czarnik T (2007). Noninvasive measurement of intracranial pressure: is it possible?. J. Trauma.

[7] Levinsky A, Papyan S, Weinberg G, Stadheim T, Eide PK (2016). Non-invasive estimation of static and pulsatile intracranial pressure from transcranial acoustic signals. Med. Eng. Phys..

[8] Avezaat CJ, van Eijndhoven JH, Wyper DJ (1979). Cerebrospinal fluid pulse pressure and intracranial volume-pressure relationships. J. Neurol. Neurosurg. Psychiatry.

[9] Takizawa H, Gabra-Sanders T, Miller JD (1987). Changes in the cerebrospinal fluid pulse wave spectrum associated with raised intracranial pressure. Neurosurgery.

[10] Eide PK (2016). The correlation between pulsatile intracranial pressure and indices of intracranial pressure-volume reserve capacity: results from ventricular infusion testing. J. Neurosurg..

[11] Kashif FM, Verghese GC, Novak V, Czosnyka M, Heldt T (2012). Model-based noninvasive estimation of intracranial pressure from cerebral blood flow velocity and arterial pressure. Science translational medicine.

[12] Schmidt B (1998). A method for a simulation of continuous intracranial pressure curves. Comput. Biomed. Res..

[13] Schmidt B, Czosnyka M, Klingelhofer J (2002). Clinical applications of a non-invasive ICP monitoring method. Eur. J. Ultrasound.

[14] Kim MO, Eide PK, O’Rourke MF, Adji A, Avolio AP (2016). Intracranial Pressure Waveforms are More Closely Related to Central Aortic than Radial Pressure Waveforms: Implications for Pathophysiology and Therapy. Acta neurochirurgica. Supplement.

[15] Nichols, W.W., O’Rourke, M.F., Vlachopoulos, C. Pressure pulse waveform analysis. In *McDonald’s blood flow in arteries. Theoretical, experimental and clinical priniciples*. 595–741 (Hodder Arnold, London, UK, 2011).

[16] Eide PK (2011). Cardiac output in idiopathic normal pressure hydrocephalus: association with arterial blood pressure and intracranial pressure wave amplitudes and outcome of shunt surgery. Fluids and barriers of the CNS.

[17] Eide PK (2006). A new method for processing of continuous intracranial pressure signals. Med. Eng. Phys..

[18] Holm S, Eide PK (2009). Impact of sampling rate for time domain analysis of continuous intracranial pressure (ICP) signals. Med. Eng. Phys..

[19] Gao M (2016). A Simple Adaptive Transfer Function for Deriving the Central Blood Pressure Waveform from a Radial Blood Pressure Waveform. Scientific reports.

[20] Bendat, J. S. & Piersol, A. G. Engineering applications of correlation and spectral analysis. *New York, Wiley-Interscience, 1980. 315 p*. (1980).

[21] Eide PK, Holm S, Sorteberg W (2012). Simultaneous monitoring of static and dynamic intracranial pressure parameters from two separate sensors in patients with cerebral bleeds: comparison of findings. Biomedical engineering online.

[22] Eide PK, Sorteberg W (2010). Diagnostic intracranial pressure monitoring and surgical management in idiopathic normal pressure hydrocephalus: a 6-year review of 214 patients. Neurosurgery.

[23] Karamanoglu M, O’Rourke MF, Avolio AP, Kelly RP (1993). An analysis of the relationship between central aortic and peripheral upper limb pressure waves in man. Eur. Heart J..

[24] Chen CH (1997). Estimation of central aortic pressure waveform by mathematical transformation of radial tonometry pressure. Validation of generalized transfer function. Circulation.

[25] Fetics B, Nevo E, Chen CH, Kass DA (1999). Parametric model derivation of transfer function for noninvasive estimation of aortic pressure by radial tonometry. IEEE Trans. Biomed. Eng..

[26] Eide PK, Egge A, Due-Tonnessen BJ, Helseth E (2007). Is intracranial pressure waveform analysis useful in the management of pediatric neurosurgical patients?. Ped Neurosurg.

[27] Eide PK (2011). A randomized and blinded single-center trial comparing the effect of intracranial pressure and intracranial pressure wave amplitude-guided intensive care management on early clinical state and 12-month outcome in patients with aneurysmal subarachnoid hemorrhage. Neurosurgery.

[28] Eide PK, Sorteberg W (2016). Outcome of surgery for idiopathic normal pressure hydrocephalus: Role of preoperative static and pulsatile intracranial pressure. World neurosurgery.

[29] Ragauskas A (2012). Clinical assessment of noninvasive intracranial pressure absolute value measurement method. Neurology.

