# Supplementary material for: Non-invasive Estimation of the Intracranial Pressure Waveform from the Central Arterial Blood Pressure Waveform in Idiopathic Normal Pressure Hydrocephalus Patients
Source: Sci Rep. 2018 Mar 16;8:4714. doi: 10.1038/s41598-018-23142-7 (PMC5856800; doi:10.1038/s41598-018-23142-7)
Supplement: Supplementary file 1 — Supplementary Information [file 41598_2018_23142_MOESM1_ESM.pdf]

Non-invasive Estimation of the Intracranial Pressure Waveform from the Central Arterial Blood Pressure Waveform in Idiopathic Normal Pressure Hydrocephalus Patients

Karen Brastad Evensen[1,2], Michael O'Rourke[3], Fabrice Prieur[1], Sverre Holm[1], Per Kristian Eide[2,4,*]

[1]Department of Informatics, University of Oslo, Oslo, Norway; [2]Department of Neurosurgery, Oslo University Hospital - Rikshospitalet, Oslo, Norway; [3]Department of Cardiology , St Vincent's Clinic, University of New South Wales/VCCRI, Sydney , Australia; [4]Institute of Clinical Medicine, Faculty of Medicine, University of Oslo, Oslo, Norway.

***Corresponding author:**

Email: p.k.eide@medisin.uio.no (PKE)

Suppl. Table 1. Information about individual patients

PatID	Age (yrs)	Gender	BMI (kg/m^2)		Mean BP (Radial artery)	Mean ICP	Mean CPP
1	79	M	20		96.1 ± 7.0	3.3 ± 4.1	92.9 ± 8.0
2	79	F	24.5		101.1 ± 11.8	5.7 ± 3.5	95.4 ± 12.6
3	77	M	23.1		112.3 ± 8.6	0.2 ± 3.2	112.1 ± 8.7
4	82	M	28.4		95.2 ± 12.7	1.8 ± 3.0	93.4 ± 11.5
5	78	F	27.2		82.4 ± 7.0	4.5 ± 3.6	77.9 ± 7.5
6	71	M	26.5		88.1 ± 7.0	2.6 ± 3.3	85.5 ± 8.0
7	74	M	24.7		85.3 ± 8.8	0.7 ± 3.5	84.6 ± 8.4
8	73	M	24		97.0 ± 12.8	0.5 ± 3.6	96.5 ± 11.5
9	75	M	25.2		96.2 ± 17.0	3.5 ± 2.8	92.7 ± 18.6
10	79	F	15.1		96.1 ± 14.1	-0.1 ± 2.8	96.1 ± 14.0
11	66	F	29.4		87.2 ± 10.5	2.1 ± 3.2	85.1 ± 11.5
12	78	F	31.3		99.6 ± 11.1	8.0 ± 4.4	91.6 ± 9.8
13	74	M	27.9		89.5 ± 8.9	9.5 ± 3.8	80.0 ± 7.3
14	70	F	17.8		97.6 ± 6.8	3.3 ± 4.0	94.3 ± 6.7
15	76	M	21.2		103.2 ± 11.7	6.3 ± 6.1	96.9 ± 13.6
16	64	F	21.2		83.7 ± 8.5	8.0 ± 4.6	75.7 ± 12.2
17	79	M	25.9		101.4 ± 16.3	6.2 ± 3.3	95.3 ± 17.4
18	75	M	22.3		83.2 ± 8.6	2.8 ± 4.3	80.4 ± 9.2
19	66	F	22.7		84.8 ± 9.0	5.4 ± 4.0	79.4 ± 9.5
20	77	F	28.7		89.7 ± 7.6	4.2 ± 4.4	85.5 ± 6.5
21	79	F	24.2		100.8 ± 7.0	8.2 ± 4.0	92.6 ± 7.9
22	80	M	25.4		95.4 ± 8.5	0.1 ± 3.8	95.4 ± 9.9
23	75	F	21.5		91.6 ± 8.7	6.7 ± 3.5	84.9 ± 9.8
24	75	F	19.6		102.2 ± 12.1	5.1 ± 4.7	97.1 ± 12.4
25	74	M	21.7		108.1 ± 10.0	0.4 ± 5.6	107.7 ± 11.6
26	83	F	24.4		88.4 ± 8.9	-0.6 ± 3.5	89.0 ± 9.9
27	84	F	22		98.8 ± 14.3	2.7 ± 3.8	96.0 ± 15.2
28	78	F	23.9		78.0 ± 7.4	2.5 ± 2.2	75.5 ± 8.5
29	53	M	29.2		72.5 ± 4.5	9.8 ± 3.2	62.7 ± 5.8
**AVG ± STD**	**74.9 ± 6.4**		**24.1 ± 3.7**		**93.3 ± 9.1**	**3.9 ± 3.0**	**89.4 ± 10.1**

Suppl. Table 2. Results of estimating pulsatile ICP based on TF from each individual (Intra-patient approach)

PatID	N 6-sec time windows	Original invasive pulsatile ICP				Absolute differences between original and estimated non-invasive pulsatile ICP		
		MWA	MWRT	MWRTC		MWA	MWRT	MWRTC
1	4,803	3.3 ± 0.7	0.24 ± 0.02	13.9 ± 2.6		1.4 ± 0.7	0.06 ± 0.04	7.7 ± 2.8
2	6,319	7.7 ± 1.8	0.23 ± 0.01	34.4 ± 7.6		4.4 ± 2.0	0.07 ± 0.03	22.7 ± 8.7
3	5,212	4.0 ± 0.8	0.27 ± 0.02	14.7 ± 2.8		2.2 ± 0.8	0.03 ± 0.03	8.5 ± 2.9
4	5,795	4.8 ± 0.7	0.19 ± 0.04	29.4 ± 7.4		1.3 ± 0.9	0.06 ± 0.05	14.2 ± 9.4
5	4,481	4.5 ± 1.5	0.27 ± 0.03	17.3 ± 5.6		1.4 ± 1.3	0.06 ± 0.03	7.1 ± 5.6
6	4,980	5.4 ± 1.6	0.30 ± 0.02	18.4 ± 5.2		1.4 ± 1.3	0.01 ± 0.01	5.0 ± 4.5
7	4,976	5.8 ± 1.0	0.24 ± 0.03	24.9 ± 4.0		1.0 ± 0.9	0.01 ± 0.02	4.6 ± 4.1
8	5,549	4.0 ± 0.7	0.27 ± 0.02	15.0 ± 2.5		0.8 ± 0.6	0.02 ± 0.02	2.2 ± 1.9
9	4,942	3.5 ± 0.7	0.27 ± 0.03	13.1 ± 3.2		1.4 ± 0.7	0.04 ± 0.03	6.2 ± 2.9
10	8,154	8.5 ± 2.0	0.27 ± 0.02	31.6 ± 6.9		2.1 ± 1.7	0.02 ± 0.02	8.2 ± 7.0
11	7,096	4.1 ± 1.2	0.27 ± 0.03	15.6 ± 5.1		1.7 ± 1.2	0.05 ± 0.04	7.9 ± 5.4
12	1,672	3.2 ± 1.0	0.29 ± 0.03	11.3 ± 3.8		1.6 ± 1.1	0.08 ± 0.04	7.3 ± 4.0
13	4,813	7.3 ± 1.2	0.29 ± 0.02	24.7 ± 4.6		5.2 ± 1.4	0.05 ± 0.03	19.4 ± 5.0
14	4,671	5.9 ± 1.4	0.28 ± 0.01	21.1 ± 4.8		1.9 ± 1.3	0.08 ± 0.03	10.3 ± 4.9
15	4,276	4.0 ± 1.5	0.21 ± 0.04	22.3 ± 9.9				
16	5,638	4.2 ± 1.0	0.25 ± 0.04	17.5 ± 4.8		1.1 ± 1.1	0.06 ± 0.05	7.3 ± 5.0
17	2,302	3.3 ± 0.6	0.17 ± 0.06	24.4 ± 7.8		2.2 ± 0.6	0.09 ± 0.06	19.3 ± 7.7
18	4,958	4.2 ± 0.9	0.23 ± 0.05	20.0 ± 5.4		0.9 ± 0.8	0.04 ± 0.03	5.2 ± 4.4
19	2,063	3.3 ± 0.5	0.17 ± 0.02	19.6 ± 2.2		1.8 ± 0.7	0.06 ± 0.05	12.3 ± 3.6
20	5,435	9.8 ± 2.3	0.33 ± 0.02	30.2 ± 6.7		2.0 ± 2.0	0.01 ± 0.01	6.9 ± 6.5
21	3,977	7.0 ± 1.4	0.25 ± 0.01	28.0 ± 5.7		2.1 ± 1.5	0.04 ± 0.02	10.6 ± 6.3
22	6,100	3.2 ± 1.3	0.20 ± 0.06	16.7 ± 5.1		1.1 ± 1.0	0.07 ± 0.06	6.6 ± 4.2
23	5,805	6.0 ± 1.6	0.29 ± 0.02	20.7 ± 4.9		1.4 ± 1.1	0.02 ± 0.02	4.7 ± 3.8
24	4,834	6.4 ± 2.2	0.28 ± 0.02	22.5 ± 7.2		3.5 ± 2.6	0.08 ± 0.04	16 ± 7.9
25	3,672	3.7 ± 0.8	0.24 ± 0.05	17.3 ± 4.9		2.3 ± 0.9	0.08 ± 0.06	12.7 ± 5.1
26	1,674	4.0 ± 0.8	0.27 ± 0.02	14.9 ± 2.8		1.8 ± 0.8	0.08 ± 0.03	9.1 ± 3.0
27	5,570	5.5 ± 1.1	0.26 ± 0.02	21.6 ± 4.4		2.7 ± 1.3	0.08 ± 0.04	13.4 ± 5.0
28	2,785	3.1 ± 0.7	0.27 ± 0.02	11.7 ± 2.5		1.6 ± 0.7	0.10 ± 0.03	7.7 ± 2.5
29	4,960	4.9 ± 1.5	0.22 ± 0.05	23.7 ± 5.5		1.0 ± 1.1	0.06 ± 0.05	7.7 ± 6.3
**AVG ± STD**		**5.0 ± 1.8**	**0.25 ± 0.04**	**20.6 ± 6.1**		**1.9 ± 1.0**	**0.05 ± 0.03**	**9.7 ± 5.0**
**SUM**	**137,512**							

Suppl. Table 3. Results of estimating pulsatile ICP based on the same TF applied to all individuals (Inter-patient approach)

PatID	N 6-sec time windows	Original invasive pulsatile ICP				Absolute differences between original ICP and estimated non-invasive pulsatile ICP		
		MWA	MWRT	MWRTC		MWA	MWRT	MWRTC
1	4,803	3.3 ± 0.7	0.24 ± 0.02	13.9 ± 2.6		0.9 ± 1.0	0.10 ± 0.06	5.2 ± 4.0
2	6,319	7.7 ± 1.8	0.23 ± 0.01	34.4 ± 7.6		4.5 ± 1.8	0.05 ± 0.03	22.3 ± 8.0
3	5,212	4.0 ± 0.8	0.27 ± 0.02	14.7 ± 2.8		1.0 ± 0.7	0.04 ± 0.02	2.5 ± 2.0
4	5,795	4.8 ± 0.7	0.19 ± 0.04	29.4 ± 7.4		0.9 ± 0.8	0.07 ± 0.04	9.8 ± 7.8
5	4,481	4.5 ± 1.5	0.27 ± 0.03	17.3 ± 5.6		1.3 ± 1.2	0.03 ± 0.02	5.9 ± 4.9
6	4,980	5.4 ± 1.6	0.30 ± 0.02	18.4 ± 5.2		1.4 ± 1.5	0.02 ± 0.02	5.3 ± 5.3
7	4,976	5.8 ± 1.0	0.24 ± 0.03	24.9 ± 4.0		0.9 ± 0.8	0.04 ± 0.04	6.1 ± 4.7
8	5,549	4.0 ± 0.7	0.27 ± 0.02	15.0 ± 2.5		1.3 ± 0.7	0.02 ± 0.02	3.6 ± 2.2
9	4,942	3.5 ± 0.7	0.27 ± 0.03	13.1 ± 3.2		0.9 ± 0.7	0.04 ± 0.03	3.3 ± 2.9
10	8,154	8.5 ± 2.0	0.27 ± 0.02	31.6 ± 6.9		3.9 ± 2.2	0.05 ± 0.03	16.8 ± 7.8
11	7,096	4.1 ± 1.2	0.27 ± 0.03	15.6 ± 5.1		0.9 ± 0.7	0.03 ± 0.02	3.7 ± 3.7
12	1,672	3.2 ± 1.0	0.29 ± 0.03	11.3 ± 3.8		1.7 ± 1.2	0.04 ± 0.03	5.5 ± 3.8
13	4,813	7.3 ± 1.2	0.29 ± 0.02	24.7 ± 4.6		2.6 ± 1.3	0.02 ± 0.02	9.4 ± 5.0
14	4,671	5.9 ± 1.4	0.28 ± 0.01	21.1 ± 4.8		1.8 ± 1.2	0.04 ± 0.02	7.7 ± 4.5
15	4,276	4.0 ± 1.5	0.21 ± 0.04	22.3 ± 9.9		1.3 ± 0.9	0.12 ± 0.04	11.4 ± 8.6
16	5,638	4.2 ± 1.0	0.25 ± 0.04	17.5 ± 4.8		1.2 ± 1.0	0.05 ± 0.04	7.2 ± 4.9
17	2,302	3.3 ± 0.6	0.17 ± 0.06	24.4 ± 7.8		1.0 ± 0.7	0.14 ± 0.06	16.2 ± 9.0
18	4,958	4.2 ± 0.9	0.23 ± 0.05	20.0 ± 5.4		0.8 ± 0.7	0.05 ± 0.05	4.9 ± 4.8
19	2,063	3.3 ± 0.5	0.17 ± 0.02	19.6 ± 2.2		1.7 ± 0.7	0.09 ± 0.04	12.9 ± 2.9
20	5,435	9.8 ± 2.3	0.33 ± 0.02	30.2 ± 6.7		2.0 ± 2.0	0.01 ± 0.01	6.9 ± 6.5
21	3,977	7.0 ± 1.4	0.25 ± 0.01	28.0 ± 5.7		2.9 ± 1.5	0.05 ± 0.02	13.8 ± 6.1
22	6,100	3.2 ± 1.3	0.20 ± 0.06	16.7 ± 5.1		1.1 ± 0.8	0.08 ± 0.06	5.4 ± 4.4
23	5,805	6.0 ± 1.6	0.29 ± 0.02	20.7 ± 4.9		1.7 ± 1.3	0.04 ± 0.02	7.1 ± 4.8
24	4,834	6.4 ± 2.2	0.28 ± 0.02	22.5 ± 7.2		2.0 ± 1.9	0.05 ± 0.02	7.9 ± 7.2
25	3,672	3.7 ± 0.8	0.24 ± 0.05	17.3 ± 4.9		1.0 ± 0.6	0.08 ± 0.05	4.8 ± 4.7
26	1,674	4.0 ± 0.8	0.27 ± 0.02	14.9 ± 2.8		0.6 ± 0.5	0.03 ± 0.02	2.4 ± 2.0
27	5,570	5.5 ± 1.1	0.26 ± 0.02	21.6 ± 4.4		2.9 ± 1.3	0.06 ± 0.03	13.1 ± 4.9
28	2,785	3.1 ± 0.7	0.27 ± 0.02	11.7 ± 2.5		0.9 ± 0.7	0.07 ± 0.03	4.3 ±2.8
29	4,960	4.9 ± 1.5	0.22 ± 0.05	23.7 ± 5.5		2.3 ± 1.4	0.07 ± 0.06	14.5 ± 6.0
**AVG ± STD**		**5.0 ± 1.8**	**0.25 ± 0.04**	**20.6 ± 6.1**		**1.6 ± 1.0**	**0.05 ± 0.03**	**8.3 ± 4.9**
**SUM**	**137,512**							
